# Cardioplegic Machine Perfusion of Hearts Donated after Circulatory Death

**DOI:** 10.1007/s12265-025-10694-z

**Published:** 2025-09-08

**Authors:** Lars Saemann, Kristin Wächter, Anne Großkopf, Sabine Pohl, Adrian-Iustin Georgevici, Fabio Hoorn, Sevil Korkmaz-Icöz, Matthias Karck, Andreas Simm, Gábor Szabó

**Affiliations:** 1 Department of Cardiac Surgery, University Hospital Halle (Saale), University of Halle, Ernst-Grube-Straße 40, 06120 Halle (Saale), Germany; 2 Department of Cardiac Surgery, University Hospital Heidelberg, 69120 Heidelberg, Germany

**Keywords:** Donation after circulatory death, Machine perfusion, Heart transplantation, Custodiol-N, HTK-N, Del Nido, Longevity, Ageing, Cardioplegia

## Abstract

We compared the effects of ex-vivo machine perfusion (EVMP) of hearts donated after circulatory death (DCD) with the single-shot solutions HTK-N and Del Nido cardioplegia (DNC) on left-ventricular (LV) contractility and myocardial microcirculation. In a DCD pig model, hearts were maintained by EVMP with hypothermic, oxygenated HTK-N (DCD-HTK-N; N = 8) or DNC (DCD-DNC; *N* = 8) followed by reperfusion with blood, including assessment of contractility and microcirculation with Laser-Doppler-Flow (LDF). We performed transcriptomics using microarrays. In DCD-HTK-N, the ESP, dp/dt_max_ and dp/dt_min_ were significantly higher (*p* < 0.05) compared to DCD-DNC. Relative LDF was higher in DCD-HTK-N vs. DCD-DNC. Pathways related to inflammatory mediators, cAMP, ion channels, intracellular signaling, and cell death were regulated differently. In DCD-HTK-N, longevity-associated pathways were up-, and ageing-associated pathways were downregulated. EVMP of DCD hearts with HTK-N results in a superior LV function, microcirculation, and regulation of pathways with short- and long-term relevance compared to DNC.

## Introduction

The transplantation of hearts donated from circulatory death (DCD) increases the number of heart transplantations (HTX). Donor heart maintenance during transportation is a driving factor for short- and long-term graft survival. DCD-hearts are exposed to warm ischemia before procurement; therefore, the appropriate preservation method is of even higher importance to prevent further graft injury[1]. Currently, EVMP with normothermic, oxygenated blood is the gold standard for the maintenance of DCD cardiac allografts[2]. However, some case reports were published describing the use of hypothermic, cardioplegic machine perfusion using the XVIVO® perfusion system and preservation solution for donor heart maintenance – also in the DCD setting[3]. Besides these case reports, we also showed in preclinical studies that hypothermic EVMP with fully crystalloid solutions, such as histidine-tryptophane-ketoglutarate-N (HTK-N), seems to be effective in maintaining and partially reconditioning left-ventricular contractility of the DCD-cardiac allograft [4–6]. Considering the XVIVO® reports, and our preclinical research results, continuous cardioplegic perfusion is a highly innovative and important preservation method in cardiac transplantology and follows these main principles:

1. Decreasing the myocardial metabolism by maintaining cardioplegic cardiac arrest and hypothermia
2. Meeting the remaining metabolic demand of the heart by providing oxygen and other nutrients through the circulating solution
3. Reducing an initial reperfusion injury that may occur already after reperfusion in the EVMP device due to the oxygenated perfusate
4. Local removal and dilution of metabolites
5. Preventing a second reperfusion injury after transplantation and final reperfusion in the recipient and thereby promoting functional integrity in both the myocardium and the coronary macro- and microvasculature

Compared to HTK-N, the XVIVO® concept follows the principle of adding erythrocytes as physiological oxygen carriers to the preservation solution for a better supply of cardiomyocytes. However, the XVIVO® solution is still part of clinical studies and not freely available. Del Nido cardioplegia (DNC) is another hypothermic preservation solution that contains erythrocytes as physiological oxygen carriers and buffer agents to prevent reperfusion injury. It is well-established in cardiac surgery and can be easily produced based on its components that are freely available on the market (Supplemental Table S1)[7].

Both solutions, HTK-N and DNC, were developed as single-shot cardioplegia. HTK-N is a crystalloid cardioplegic solution, and its cardioplegic properties are mainly based on elevated potassium and low sodium concentrations[4]. The cardioplegic properties of DNC are also based on high potassium concentrations, in addition to sodium-channel blocking^7^. However, neither solution was compared with the other for the maintenance of donor hearts. Thus, in a porcine model, we investigated the preservation of DCD hearts by hypothermic oxygenated EVMP with HTK-N or DNC and compared ventricular contractility, coronary macro- and microcirculation, and underlying molecular mechanisms on the transcriptome level.

## Material and Methods

### Animals and Anesthesia

The experiments were approved by the local ethics committee for animal experimentation. We sedated healthy pigs (40–45 kg bodyweight) with an intramuscular injection of ketamine (22.5 mg/kg; Bremer Pharma, Warburg, Germany) and midazolam (0.375 mg/kg; Hameln pharma plus, Hameln, Germany) as described previously [8]. For maintenance of anesthesia, we continuously delivered pentobarbital-sodium intravenously through the ear vein (15 mg/kg/h; Boehringer Ingelheim Vetmedicia, Ingelheim, Germany). We used Dipidolor (1.125 mg/kg/h; Piramal Critical Care, Voorschoten, Netherlands) for analgesia. The partial pressure of oxygen (paO_2_) was adjusted to 200 mmHg, and the partial pressure of carbon dioxide (paCO_2_) was adjusted to 35–45 mmHg. We monitored the blood pressure and performed blood sampling through arterial and venous vascular access in the femoral artery and vein. We opened the chest by median sternotomy, exposed the heart, and injected heparin (LEO Pharma, Neuisenburg, Germany) intravenously.

### Study Groups

**Fig. 1:**
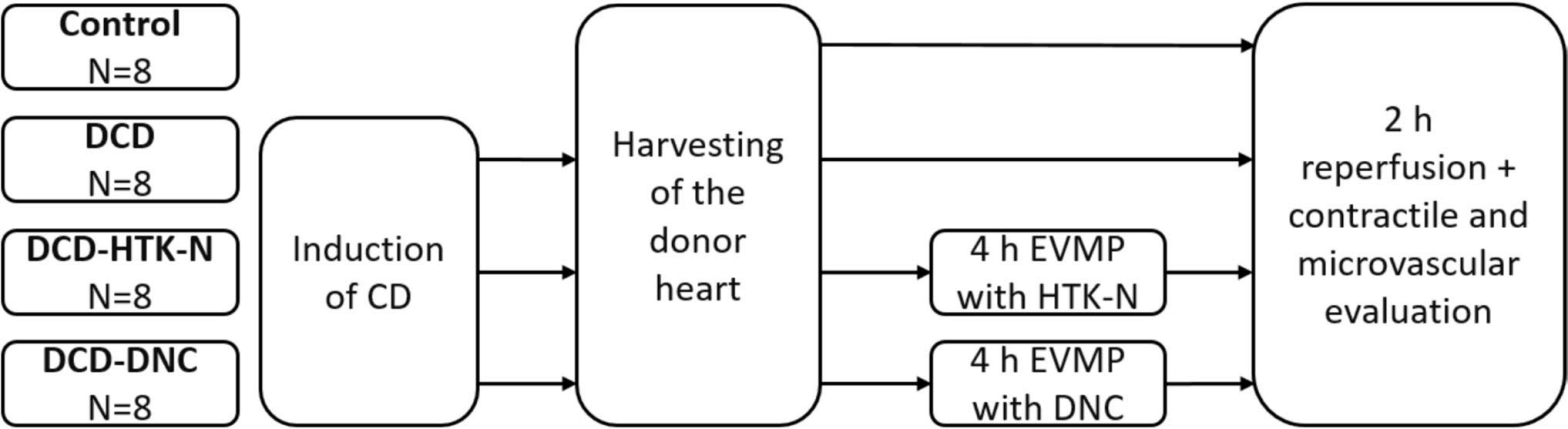
Groups. CD: Circulatory death. DCD: Donation after circulatory death. EVMP: Ex-vivo machine perfusion

We opened the chest by median sternotomy, exposed the heart, and injected heparin (LEO Pharma, Neuisenburg, Germany) intravenously. We included four groups (all groups N = 8): In a control group (Control), native control hearts were cardioplegically arrested with 2 l of cold (4°C) Custodiol® organ preservation solution (Köhler Chemie GmbH, Bensheim, Germany), harvested, and immediately assessed for contractile and microvascular function for 2 h (Fig. 1). In a DCD group (DCD), circulatory death was induced, and the hearts were evaluated immediately after harvesting. In two other groups, we maintained the DCD hearts for 4 h by hypothermic (4 °C) oxygenated EVMP with HTK-N (DCD-HTK-N) or DNC (DCD-DNC) followed by 2 h of reperfusion including functional evaluation to mimic transplantation.

### Donation after Circulatory Death Model and Machine Perfusion

We induced circulatory death by the termination of mechanical ventilation [4]. Within the subsequent 30 min, we collected blood to prepare the DNC solution (Table S1) and harvested the heart. After a total warm ischemic time of 30 min, we flushed the DCD hearts with 2 l of cold (4°C) Custodiol® organ preservation solution. The perfusion system consisted of a hollow-fiber membrane oxygenator with an integrated heat exchanger, hard-shell reservoir, roller pump, and tubing. In both groups, HTK-N and DNC, the hearts were perfused with 20–25 mmHg perfusion pressure through the ascending aorta. During EVMP, the hearts were immersed in the perfusion solution to ensure adequate cooling.

### Reperfusion with Blood and Functional Analysis

After 4 h of EVMP with HTK-N or DNC, the hearts were reperfused with fresh blood with added heparin (5.000 iU), sodium chloride, magnesium chloride, glucose, sodium-prednisolone, sodium hydrogen carbonate, and mannitol. The partial pressure of oxygen was adjusted to 180–200 mmHg, and the partial pressure of carbon dioxide was adjusted to 35–45 mmHg. The pH was adjusted to 7.35–7.45. We applied a pressure-controlled perfusion regimen with a perfusion pressure of 60 mmHg. We measured arterial and venous blood gas every 30 min with a point-of-care analyzer (RAPID Point 500, Siemens). The contractility was measured using a left ventricular balloon catheter inserted through the mitral valve. During contractility measurement, the coronary perfusion pressure was 60 mmHg. During pressure-contractility matching, the contractility was measured at a constant left-ventricular filling volume of 10 ml. During reperfusion, we assessed the end-systolic (ESP) and end-diastolic pressure (EDP), maximal slope of pressure increment (dp/dt_max_), and minimum slope of pressure decrement (dp/dt_min_) at different ventricular fillings and perfusion pressures, as measures of ventricular contractility during systole and relaxation during diatole. We measured myocardial microcirculation using a Laser-Doppler-Perfusion (LDP) system (PeriFlux 5000, Perimed, Järfälla-Stockholm, Sweden) and needle probe (Perimed, Järfälla-Stockholm, Sweden)[9].

### Tissue Collection

At the end of reperfusion, we flushed the hearts with an ice-cold Ringer solution. Myocardial tissue samples were immediately snap-frozen in liquid nitrogen. The LAD, including surrounding myocardial tissue, was quickly excised and placed in an ice-cold carbonized Krebs–henseleit-buffer solution. Under the microscopic vision, the LAD was carefully but quickly dissected and snap-frozen in liquid nitrogen. All tissue samples were stored at −80°C until further investigation.

### RNA Preparation

Total RNA was isolated from the left-ventricular myocardium and LAD of every pig (N = 8 per group; N = 32 in total). RNA was isolated by TRIzol (Thermo Fisher Scientific, Waltham, USA;) extraction. Therefore, the samples were homogenized using a Tissue Lyser II (Quiagen, Netherlands). Then, chloroform was added to induce phase separation. After centrifugation, the upper phase was agitated by incubation with isopropanol. Then, we pelletized the RNA by centrifugation at 4 °C and washed the pellet with NaAc. Then, the pellet was dissolved in DEPC-H_2_O. As a next step the pellet was precipitated in 99.8% ethanol over night at −20°C. After centrifugation the pellet was washed with 80% ethanol and centrifuged again. The supernate was removed. Finally, the pellet was solved in DEPC-H_2_O by incubation at 60 °C for 10 min and then processed as described in the next paragraph.

### Microarrays

First, we assessed the RNA integrity using the Bioanalyzer (2100 Bioanalyzer, Agilent, USA). We determined the RNA concentration using Nanodrop One (Thermofisher, USA). Biotin-labeled ss-cDNA was synthesized from total RNA with a GeneChip™ WT Pico Reagent Kit (Thermo Fisher Scientific, Waltham, USA), fragmented, and subsequently hybridized using porcine arrays (Thermo Fisher Scientific, USA). Afterward, the chips were washed and scanned by the Affymetrix GeneChip Scanner 7G. One control group sample was later identified as an outlier and, therefore, excluded from the analysis.

### Statistical Analysis

We performed the statistical analysis using IBM SPSS Statistics for Windows (Version 25.0, IBM Corp. Armonk, NY, USA). Results are expressed as mean ± standard error. We tested for homogeneity of variances using the Levene test. We analyzed the data using a one-way analysis of variance for multiple comparisons with Tukey adjustment of *p*-values in case of variance homogeneity and Games-Howell adjustment in case of variance inhomogeneity. A value of p < 0.05 was considered statistically significant. Heat maps and volcano plots were built using the Transcriptome Analysis Console (TAC 4.0; applied biosystems; Thermo Fisher Scientific, Waltham, MA, USA). Differentially expressed genes were displayed through fold change (upregulated > 2.0; down-regulated <  − 2.0), together with a *p*-value < 0.01 using eBayes statistics. We also performed a functional annotation analysis for the identification of pathway-based sets [10]. To identify the transcripts that might be of major importance for left-ventricular functional group differences, we performed a Kendall correlation analysis for transcripts that were regulated significantly differently between DCD-DNC and DCD-HTK-N.

## Results

### Left-Ventricular Contractility and Relaxation

**Fig. 2:**
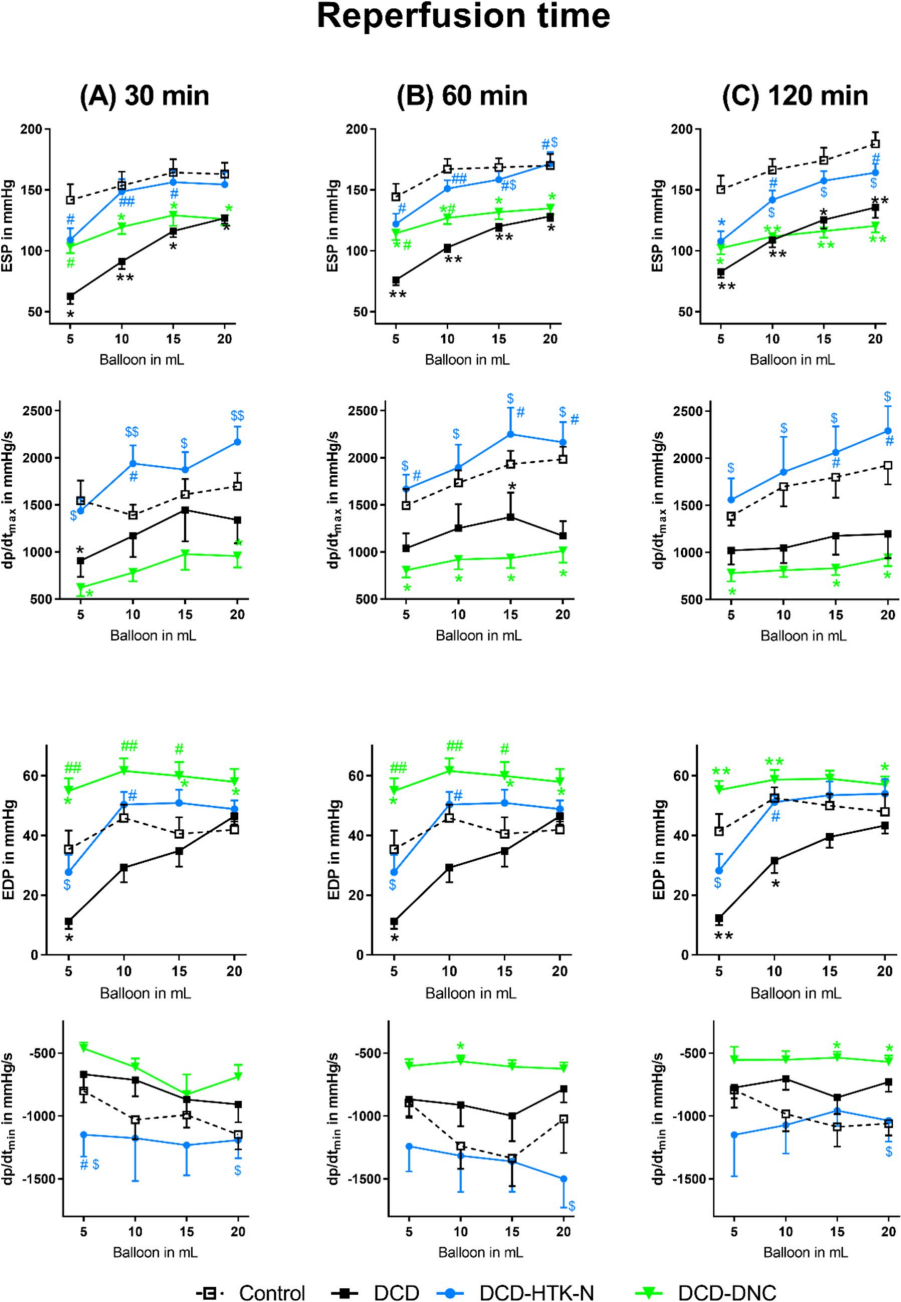
Left-ventricular contractility. After (**A**) 30 min, (**B**) 60 min and (**C**) 120 min of reperfusion. DCD: Donation after circulatory death. DNC: Del Nido cardioplegia. HTK-N: Histidine-tryptophane-ketoglutarate-N. *p < 0.05 and **p < 0.001 vs. Control. ^#^*p* < 0.05 and ^##^*p* < 0.001 vs. DCD. ^$^*p* < 0.05 and ^$$^*p* < 0.05 vs. DNC

**Fig. 3:**
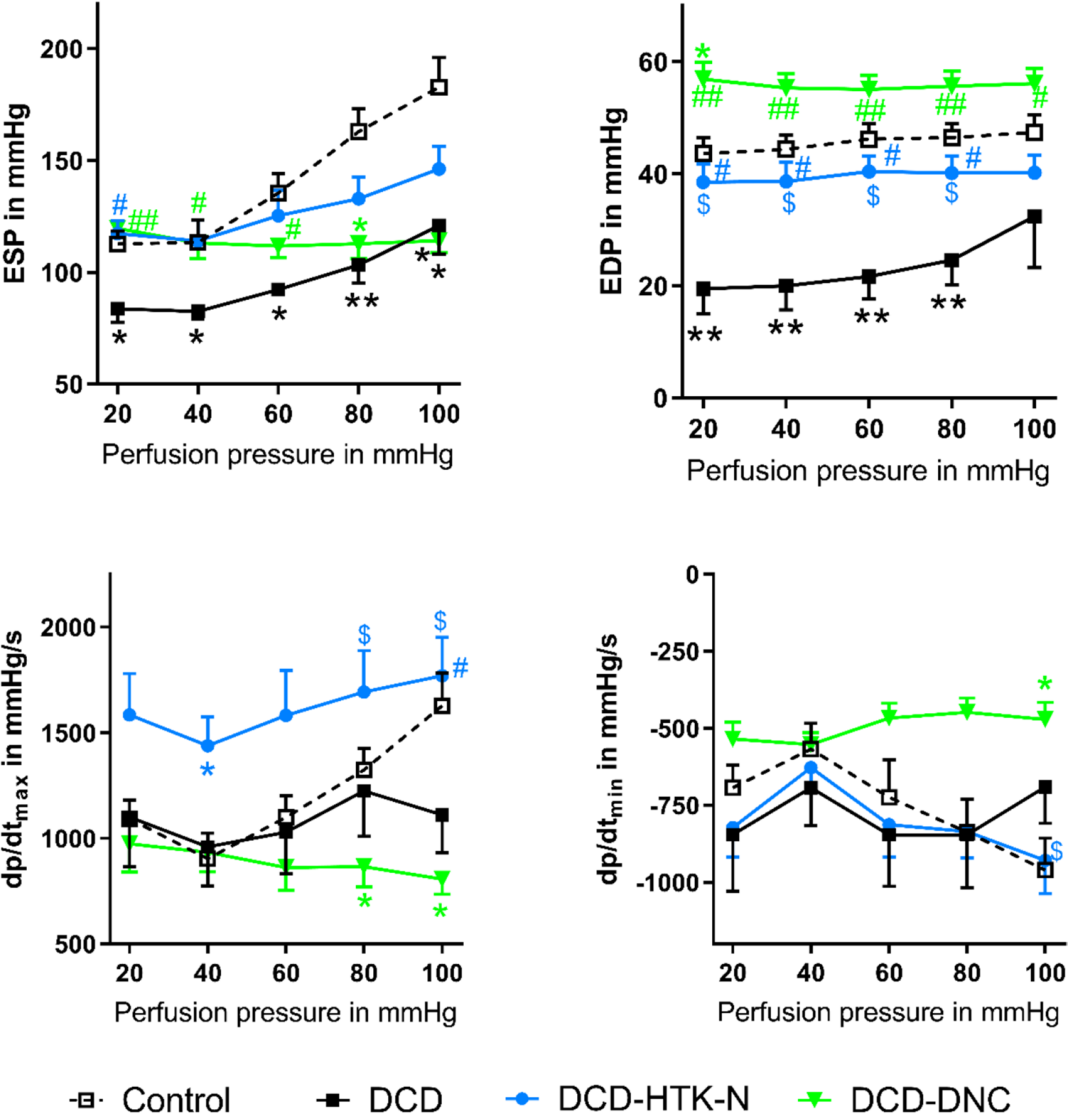
Pressure-Contractility-Matching during final reperfusion with blood after EVMP with HTK-N or DNC. CF: Coronary flow. DCD: Donation after circulatory death. DNC: Del Nido cardioplegia. EVMP: Ex-vivo machine perfusion. HTK-N: Histidine-tryptophane-ketoglutarate-N. LDP: Laser-Doppler-Perfusion. *p < 0.05 and **p < 0.001 vs. Control. ^#^*p* < 0.05 and ^##^*p* < 0.001 vs. DCD. ^$^*p* < 0.05 vs. DNC

The ESP and dp/dt_max_ decreased significantly in DCD and DCD-DNC compared to Control (Fig. 2). In DCD-HTK-N, ESP and dp/dt_max_ were significantly higher than in DCD and DCD-DNC. The EDP was significantly increased in DCD-DNC compared to Control (Fig. 2). In DCD-HTK-N, EDP and dp/dt_min_ were significantly lower than DCD-DNC. In DCD-DNC, dp/dt_min_ was significantly increased compared to Control. During PCM (Fig. 3), at low perfusion pressures, ESP was significantly higher in DCD-HTK-N and DCD–DNC compared to DCD. Nevertheless, ESP increased in all groups, except DCD-DNC, by increasing perfusion pressures. In DCD-HTK-N, dp/dt_max_ was significantly higher than in DCD and DCD-DNC, especially at high perfusion pressures. Dp/dt_min_ showed a comparable course in DCD-HTK-N and Control and was significantly higher in DCD-HTK-N compared to DCD-DNC.

### Coronary Circulation

**Fig. 4:**
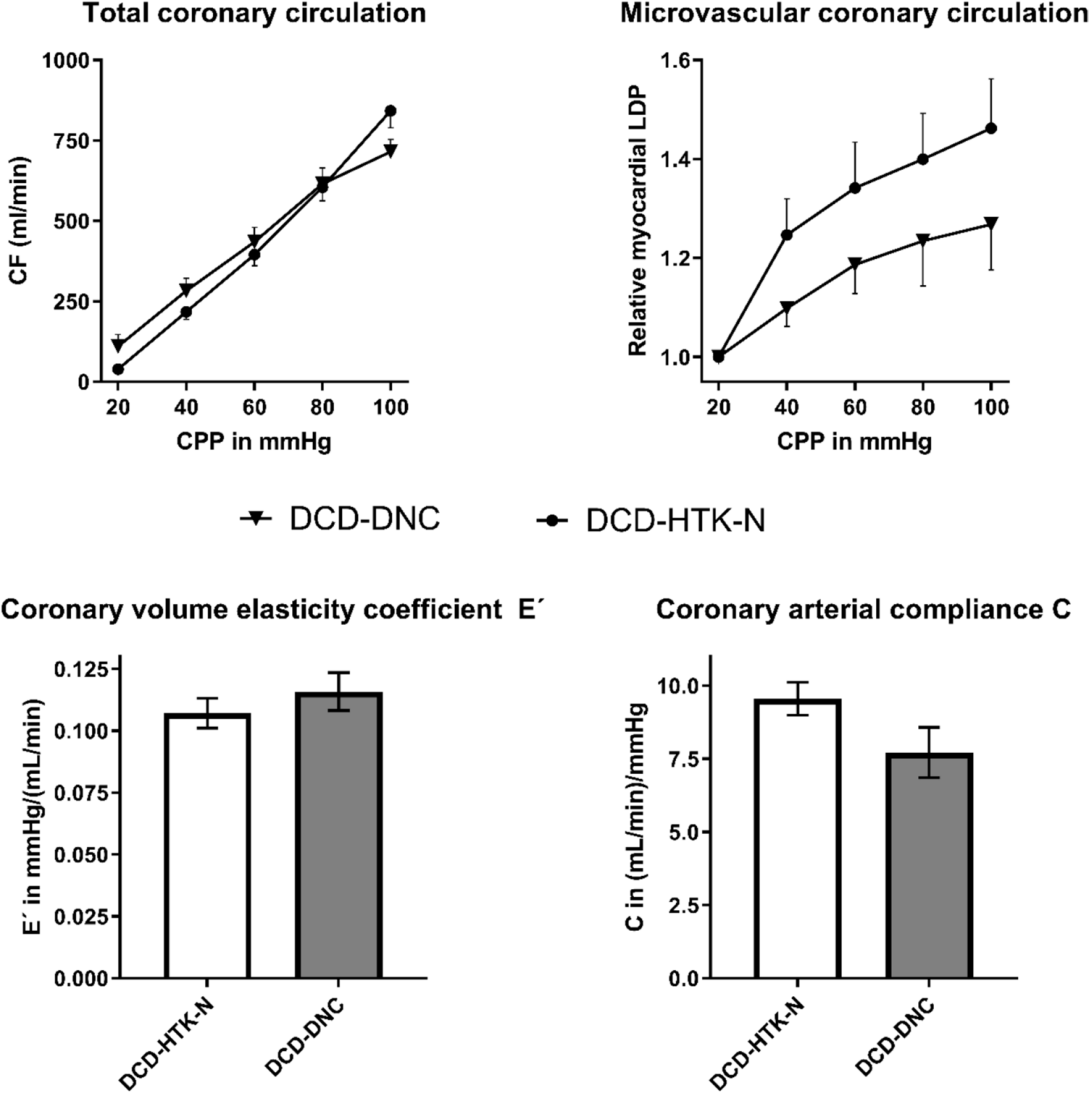
Myocardial microcirculation during final reperfusion with blood after EVMP with HTK-N or DNC. In DCD-HTK-N, LDP was measured only in *N* = 7 hearts due to detachment of the probe in one case. CF: Coronary flow. DCD: Donation after circulatory death. DNC: Del Nido cardioplegia. EVMP: Ex-vivo machine perfusion. HTK-N: Histidine-tryptophane-ketoglutarate-N. LDP: Laser-Doppler-Perfusion

The total coronary flow was comparable between DCD-HTK-N and –DNC (Fig. 4). Nevertheless, the relative LDP was increased by higher perfusion pressures in DCD-HTK-N compared to DCD-DNC. The coronary arterial compliance C was higher in DCD-HTK-N compared to DCD-DNC.

### Gene Expression

**Fig. 5:**
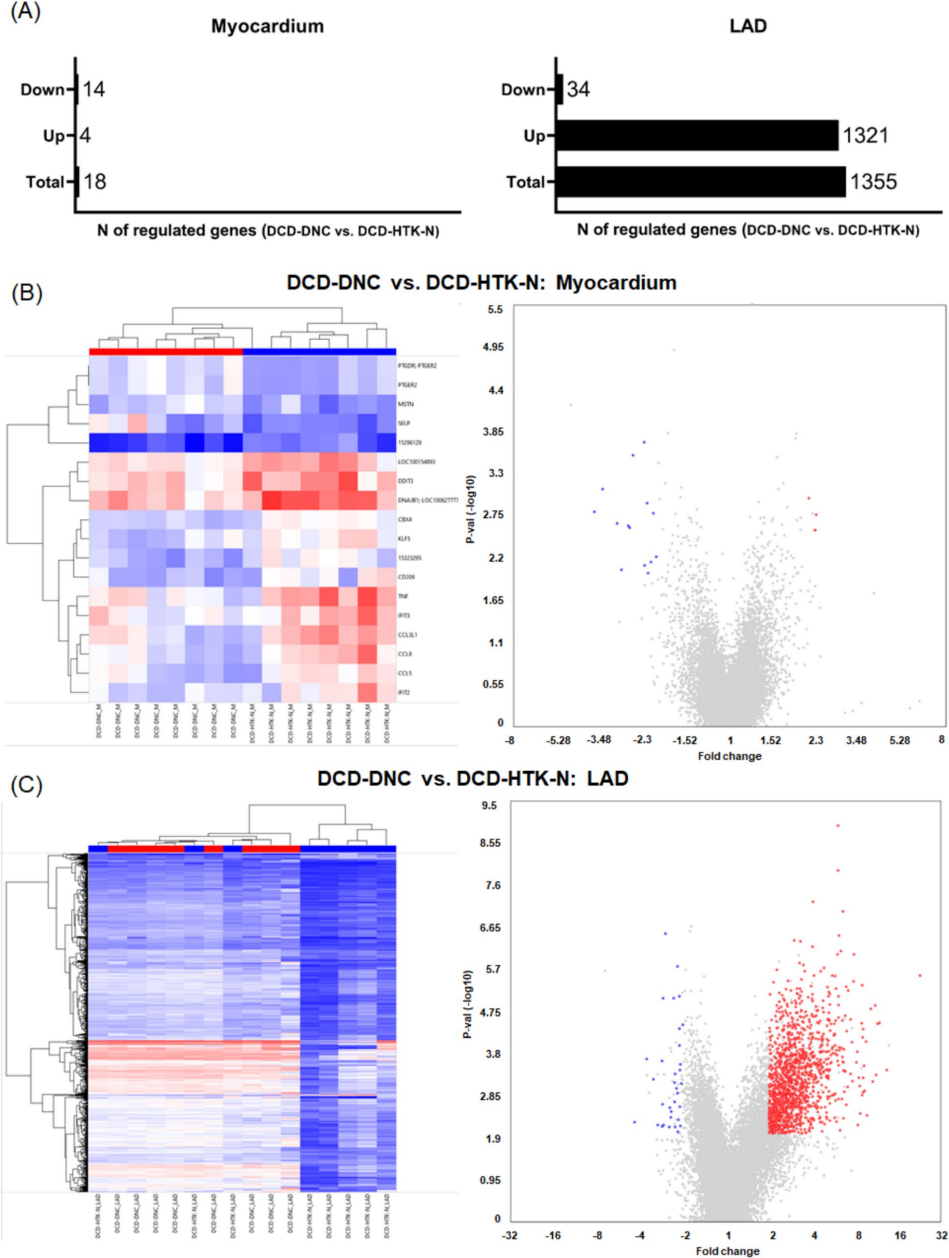
Heat maps and volcano plots. (**A**) Number of regulated genes. (**B**) Gene expression in the myocardium. (**C**) Gene expression in the LAD. DCD: Donation after circulatory death. DNC: Del Nido cardioplegia. HTK-N: Histidine-tryptophane-ketoglutarate-N. LAD: Left anterior descending coronary artery

In general, in vivo ischemia (DCD) induced a predominant downregulation of transcripts compared to the controls. Subsequently, cardioplegic EVMP reinforces this effect in the myocardium but reverses it in the LAD to different extents. In the myocardium, 14 genes were down- and 4 genes were upregulated in DCD-DNC vs. DCD-HTK-N (Fig. 5). In the LAD, a total of 1355 transcripts were significantly regulated. 34 genes were down- and 1321 genes were upregulated in DCD-DNC compared to DCD-HTK-N. The individual heart samples of each group clustered well together.

### Myocardial Pathway Analysis

EVMP can treat the heart during transportation. Thus, we investigated how both perfusion solutions affected the gene expression compared to the DCD group. In the myocardium of DCD-DNC, pathways associated with cytokine signaling, cytokine receptor CXCR3, and cytokines such as INFα/β, TNF, IL-6, IL-17, IL-18, IL-23, IL-27 were downregulated compared to DCD. Also, chemokine binding and signaling, the JAK/STAT pathway, and p53 were downregulated. Only CYSLTR1 was significantly upregulated in the myocardium in the DCD-DNC compared to the DCD group.

In the myocardium of DCD-HTK-N hearts, pathways associated with cytokine signaling involving cytokines such as INFα/β, TNF, IL-4, IL-6, IL-17, IL-18, cytokine receptor activation, and inflammatory response pathways in general were also downregulated compared to DCD hearts. Next to the chemokine binding and signaling, the JAK/STAT pathway and p53, pathways associated with ferroptosis, the complement system, and eicosanoid metabolism were additionally downregulated. The miRNA regulation of DNA damage response was upregulated.

### LAD Pathway Analysis

In the LAD, pathways involved in the electron transport chain, oxidative phosphorylation, citric acid cycle, and cellular response to starvation or stress were downregulated in the DCD-DNC compared to the DCD group. Upregulated pathways included inflammatory response, cytokines such as IL-2 to IL-7, IL-9, IL-11, IL-12, IL-17, IL-27, TNF, TGFβ, IFNα and INFγ, pathways for cytokine production, such as JAK/STAT, and elements of the immune or cytokine-mediated stress reaction such as the t-cell receptor, AP-1, MAPK signaling, P53. Other upregulated pathways were associated with vascular development, such as VEGF-VEGRFR2 signaling, angiogenesis, extracellular matrix, vascular function, such as cGMP-PKG or development in general, such as Wnt signaling, C-MYC, RAC1, focal adhesion, selenocysteine synthesis, and seleno amino acid metabolism, PI3K-Akt-mTOR, PIP3 activates Akt, FGF signaling and respective receptors FGFR, FGFR2. Additionally, pathways associated with post-translational-protein modification, senescence, and cell death, such as necroptosis and apoptosis, AGE-RAGE, and EPO, were upregulated in the LAD of DCD-DNC compared to the DCD group.

In the LAD of DCD-HTK-N hearts compared to DCD hearts, respiratory electron transport, oxidative phosphorylation, citric acid cycle, cellular response to chemical stress, and selenocysteine synthesis were downregulated. Upregulated pathways were associated with inflammatory response, MAPK signaling, and cytokines such as IL-2, IL-4, IL-6, IL-17, IL-18, TGFβ and its receptor, and NFkB. Additionally, pathways responsible for vascular development, such as VEGFA/VEGFR2, extracellular matrix, focal adhesion PI3K-Akt-mTOR signaling pathway, and Akt signaling, were upregulated. Pathways that counteract development, such as repression of WNT target genes and EGFR tyrosine kinase inhibitor, were also upregulated. Other upregulated pathways were associated with cellular response to stress, HSF-1-dependent transactivation, and HIF-1-alpha transcriptional network.

### Targeted Transcriptome Analysis

In light of the different compositions, a manual analysis of the array data was also performed to outline potential component-dependent regulations and highlight possibilities to improve preservation solutions. The prostaglandin-receptor PTGER2 (EP2) transcript was downregulated in the myocardium in HTK-N vs. DNC. PTGFR showed the same tendency. In the LAD, the receptor transcription was not altered. However, PTGIS, a prostaglandin-synthase, adenylyl cyclase 6, and beta-catenin 1 (CTNNB1) transcripts were downregulated as are cAMP-specific and dependent enzymes (PKIA, PRKAG2, PRKAR1A, PRKAR2A, PDE4D, PDE5A). Furthermore, several potassium channels had deregulated transcripts in DNC compared to Control (downregulated: HCN3, KCNA3, KCNA4, KCTD11, KCNK3), compared to DCD (downregulated: KCNK7), compared to Control and DCD (upregulated: SLC12A6, KCTD12, KCNMA1, KCNMB1, KCMF1) and compared to HTK-N (upregulated: KCTD20) which were not significantly regulated in HTK-N vs. Control or DCD. A variety of transcripts for sodium channels were deregulated similarly, with a pronounced upregulation of SCN7A in DNC vs. HTK-N. Furthermore, CLCC1, a chloride channel, was also upregulated in DNC vs. HTK-N, as was its binding partner, calreticulin. Several immune-cell-related transcripts were differently upregulated in HTK-N vs. DNC in the myocardium: CCL5, CCL8, CCL3L1, CD209, and TNF[11]. In contrast, transcripts of selectin P (CD62) and selectin e were downregulated in HTK-N compared to DCD alone and in the case of CD62 also vs. DNC in LAD and myocardium. DNC perfusion led to a more pronounced activated gene response in LAD than HTK-N (76% to 68% vs. Control; 88% to 67% vs. DCD upregulated transcripts). Within this data, biological processes of ubiquitin-mediated protein degradation and vesicular transport were differentially enriched (DNC vs. HTK-N). Of note, 52 transcripts containing"ubiquitin"in the description were regulated in DNC vs. HTK-N in LAD; the only downregulated transcript was ubiquitin B. MYSM1, a deubiquitinase of H2A, was strongly upregulated by DNC in the LAD as compared to HTK-N, but downregulated by HTK-N in the myocardium, if compared to Control. In contrast, the YOD1 deubiquitinase transcript was only regulated in the myocardium, displaying a compensatory upregulation by HTK-N. On the other hand, DNC downregulated the counteracting ubiquitin-ligase TRIM31 and showed a tendency for compensatory upregulation in LAD (HTK-N vs. DNC). A similar pattern could be found for transcripts of kinases, of which 72 were up- and 2 downregulated in DNC vs. HTK-N in LAD and phosphatases, of which 27 were up- and 0 were downregulated.

### Correlation Analysis

**Fig. 6:**
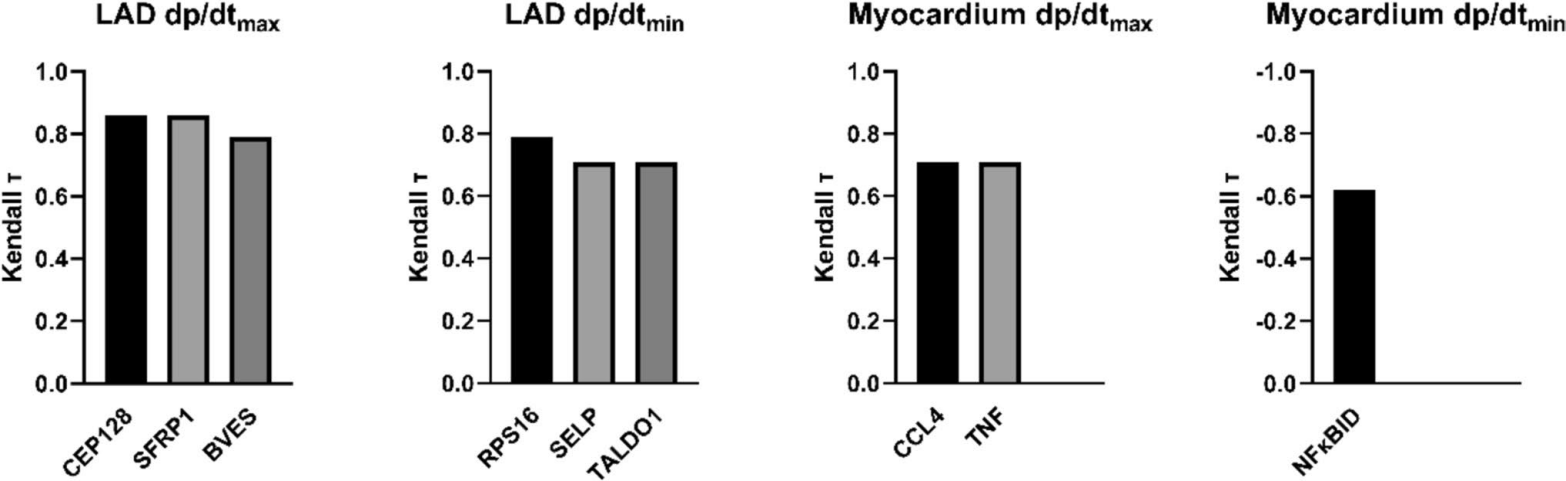
Top 3 genes that correlate with left-ventricular function after HTK-N perfusion

Figure 6 shows the top 3, or less (if less were identified), transcripts of the LAD and the myocardium that significantly correlated with the leftventricular functional parameters dp/dt_max_ and dp/dt_min_ in DCD-HTK-N. CEP128, SFRP1, and BVES in the LAD, and CCL4 and TNF significantly correlated with dp/dt_max_. RSP16, SELP, and TALDO1 in the LAD and NFκBID in the myocardium significantly correlated with dp/dt_min_.

## Discussion

The underlying reasons for the superior left-ventricular systolic and diastolic function and microvascular circulation, based on composition-related considerations and transcriptome-based findings, are diverse. Both solutions contain high potassium concentrations to initially induce a depolarizing cardiac arrest. By arrest induction/maintenance and hypothermic conditions, both solutions reduce the metabolic demand. HTK-N contains very low amounts of sodium to maintain a hyperpolarizing arrest. DNC, which is not characterized by low sodium concentrations, contains the sodium channel blocker lidocaine instead. Thus, both solutions prevent transmembrane sodium influx. However, the array data indicate that the regulation of sodium and potassium channels in DNC-perfused hearts is not only facilitated by lidocaine but strongly influenced by other components, causing an upregulation of specific channels. This regulation could be implemented by the higher ion concentrations and might lead to inferior contractile performance if the effects translate to the protein level.

The calcium concentration in both solutions is low, preventing hypercontraction. Additionally, both solutions contain magnesium as a calcium channel antagonist to reduce calcium influx from the blood during reperfusion and thereby provide better diastolic relaxation, which was only apparent in DCD-HTK-N [12]. Chloride ions increase the cellular injury after reperfusion and are reduced only in HTK-N. The chloride concentration in Plasmalyte A matches the chloride concentration in blood. Consequently, DNC does not seem to prevent chloride-mediated injury during reperfusion. The array data revealed a DNC-dependent upregulation of CLCC1, a chloride channel, and its binding partner calreticulin, presumably in response to calcium and chloride contents[13, 14].

HTK-N contains protective constituents, such as Nα-acetyl-L-histidine, which improves endothelial function after vascular ischemia/reperfusion[15], L-arginine as a substrate for the endothelial nitric oxide synthase to enhance the blood flow[16], L-alanine and L-glycine to decrease the sodium-influx[17, 18], and α-Ketoglutarate, which improves cardiac function after cardioplegic cardiac arrest[19]. The superior microvascular perfusion in DCD-HTK-N might have been caused by Nα-acetyl-L-histidine and L-arginine. Microvascular dysfunction in DCD-DNC could also have caused decreased diastolic relaxation due to potential diastolic cross-bridge cycling[20]. Deep hypothermic conditions induce cell injury by the formation of free oxygen radicals by redox-active iron ions that can be bound by the iron chelators LK614 and Desferoxamine[16].

In light of the organ performance, an upregulation of immune-cell-related transcripts is counterintuitive. However, all of these transcripts have smaller, non-significant changes in HTK-N vs. Control, thus prompting a compensatory re-upregulation in DCD-HTK-N to reestablish control levels. Additionally, much fewer pathways associated with inflammation and cytokine production were upregulated in the LAD of DCD-DNC than in DCD-HTK-N vs. DCD. Interestingly, the myocardium pathways related to cytokine production, such as JAK/STAT, were downregulated in both DCD-HTK-N and -DNC, with one exception: CYSLTR1, which is involved in the activation of inflammatory processes, was upregulated in DCD-DNC vs. DCD but not in DCD-HTK-N vs. DCD. Furthermore, only in the DCD-HTK-N myocardium, the complement system was downregulated compared to DCD.

For human hearts, distinct macrophage subsets are discussed that need to be attracted and are required for physiological cardiac functioning. E.g., CD209 is a marker for anti-inflammatory macrophage subsets[11]. Selectin transcripts, in contrast, were downregulated in DCD-HTK-N, attenuating binding capacities for immune cells and platelets and are thereby able to mirror the grade of microvascular inflammation[21]. Consequently, the downregulated selectin transcripts might also be a reason for the higher microvascular flow. P-selectin, which can be upregulated by ROS, was lower expressed in the DCD-HTK-N group, suggesting a lower ROS formation than in DNC.

The stronger upregulation of some prostaglandin-synthase-and CTNNB1-associated transcripts, as well as transcripts associated with cAMP-specific enzymes in DCD-DNC compared to -HTK-N indicates a regulation towards control levels in DCD-HTK-N while having a continued or even reinforced effect in DCD-DNC which could be part of an explanation, why DCD-DNC performed so poorly. A general broad and intense upregulation of transcripts is found in DCD-DNC, especially in the LAD. Transcripts influencing prostaglandin and cAMP-based cellular signaling, kinase, phosphatase composition, and ubiquitin-mediated protein degradation and signaling are affected. Thus, the data suggests a beginning of large-scale remodeling of the cellular protein repertoire in response to a diverse pattern of stimuli in DCD-DNC, which might be attributable to the diverse constituents of the blood component of DNC, while HTK-N perfusion showed a more distinct and balanced regulation.

Apoptosis pathways, such as p53, were downregulated in the myocardium of both groups compared to DCD but upregulated in the LAD of DCD-DNC. Necroptosis pathways were also upregulated in the LAD of DCD-DNC. Ferroptosis pathways were downregulated in the myocardium of DCD-HTK-N. Pathways associated with vascular development and development, in general, were much more upregulated in the LAD of DCD-DNC than in DCD-HTK-N vs. DCD, which might be based on less injured tissue in DCD-HTK-N. The downregulated eicosanoid metabolism-associated pathways in DCD-HTK-N align with published findings showing that many eicosanoids are associated with heart failure[22].

The upregulated HSF-1 in the LAD of DCD-HTK-N vs. DCD increases stress resistance and promotes longevity, as shown in the nematode[23]. Pathways associated with aging were upregulated in the LAD of DCD-DNC vs. DCD. The upregulated AGE-RAGE pathway in the LAD of DCD-DNC vs. DCD also promotes various pro-inflammatory processes that lead to age-associated arterial diseases[24]. The upregulation of the HIF-1-alpha transcriptional network in DCD-HTK-N and downregulation of pathways associated with cellular response to starvation in DCD-DNC suggest that the low amount of oxygen carriers in DNC might be beneficial, although functional results were inferior.

### Correlation of Transcripts with Ventricular Function

SFRP1 protects rat cardiomyoblasts from hypoxia/re-oxygenation injury, myocardial damage, early leukocyte infiltration, and apoptosis, and it increases capillary density and improves myocardial function after infarction[25]. BVES enhances the preservation of cardiomyocytes[26]. SELP might be involved in developing atherosclerosis[27] and TALDO1 in obstructive coronary artery disease [28]. NFκBID inhibits NFκB and thereby has a regulatory effect on cytokine production[29]. Considering that the expression of these transcripts in DCD-HTK-N was comparable to the Control and that they correlated significantly with the ventricular function, it suggests that they are important for contractile and relaxative performance after EVMP.

### Limitations

This work is limited by the fact that porcine and not human hearts were used for the experiments. However, porcine hearts are closely comparable to human hearts in anatomical structure and physiological function[30]. Thus, porcine hearts are commonly used for experiments on myocardial I/R and transplantation, which makes them valuable for translational research, as shown in the present study[31, 32]. Additionally, the length of the agonal phase, which occurs in DCD, is less variable in animal models compared to humans, as reported by Heinis et al. [32]. Nevertheless, this should only lead to a stabilization of results rather than a bias for interpretability and reliability.

## Conclusion

Both solutions can maintain a hypothermic cardioplegic arrest. Nevertheless, EVMP with HTK-N results in superior left-ventricular contractility, relaxation, and microcirculation. Thus, maintaining hypothermic conditions, cardiac arrest, and oxygenation are not the only driving factors for maintaining DCD hearts during EVMP. The composition of the solutions leads to differently regulated relevant pathways in the myocardium and coronary vasculature. The promotion of longevity-associated and reduction of aging- and senescence-associated pathways could also lead to superior long-term results after HTK-N perfusion and should be focused on in future studies.

### Clinical relevance

Adding blood as a component of the perfusion solution in EVMP of DCD hearts does not necessarily improve the ventricular function. Hypothermic, oxygenated EVMP with HTK-N, as compared to DNC, would most likely lead to higher ventricular contractility and thus presumably lower rates of primary graft dysfunction after HTX with DCD.

## Abbreviations

DCD: Donation from circulatory death
DNC: Del Nido cardioplegia, Del Nido cardioplegia
dp/dt_max_: Maximal slope of pressure increment
dp/dt_min_: Minimum slope of pressure decrement
EDP: End-diastolic pressure
ESP: End-systolic pressure
HTX: Heart transplantations
LDP: Laser-doppler-perfusion
LAD: Left anterior descending coronary artery

## Declarations

**Ethical Approval for Animal Study.**

All institutional and national guidelines for the care and use of laboratory animals were followed and approved by the appropriate institutional committees.
